# Inflammation and endothelial gene polymorphism are associated with ischemic stroke

**DOI:** 10.3389/fneur.2025.1465559

**Published:** 2025-02-05

**Authors:** Wanli Chen, Jie Li, Jintao Zhou, Xingyang Yi, Hong Chen

**Affiliations:** ^1^Department of Neurology, The People’s Hospital of Deyang City, Deyang, China; ^2^Department of Neurology, The Yongzhou Central Hospital, Yongzhou, China

**Keywords:** stroke, high-risk stroke population, genetic polymorphism, inflammation, endothelial function

## Abstract

**Aim:**

Evaluate the correlation between ischemic stroke and genetic variations related to inflammation and endothelial function.

**Methods:**

This was a multicenter cross-sectional research conducted in southwestern China. Residents aged ≥40 years voluntarily join in the face-to-face survey in 8 communities. 2,377 participants were at high risk of stroke, of which 429 had a previous history of ischemic stroke. We selected the 429 ischemic stroke patients as the research subjects, and adopted a 1:1 matching method to select 429 healthy people with a 2-year age difference and the same gender and hypertension as the control group. We detected genotypes of 19 variants in 10 genes related to inflammation and endothelial function. Analyze gene–gene interaction through generalized multifactor dimensionality reduction (GMDR).

**Results:**

Analysis found no statistically significant differences in age, gender, hypertension, BMI, and smoking history between ischemic stroke patients and healthy control group. Compared with the healthy group, ischemic stroke group has a higher proportion of diabetes, heart disease, dyslipidemia, stroke family history, and a higher proportion of lack of exercise. *HABP2* rs7923349, *NOS2A* rs8081248, *HABP2* rs932650 were related to stroke in univariate analysis. GMDR analysis showed significant gene–gene interactions between *HABP2* rs7923349, *HABP2* rs932650. After adjusting for covariates, high-risk interaction genotypes between these two variants were independently associated with higher stroke risk (OR, 3.578, 95% CI: 2.618–4.890, *p* < 0.001).

**Conclusion:**

This study found that specific variations in genes related to inflammation and endothelial function are associated with ischemic stroke. The high-risk interactive genotypes among *HABP2* rs7923349, *HABP2* rs932650 distinctly increased the risk of ischemic stroke.

## 1 Introduction

Stroke is one of the leading cause of death and adult disability in China (1, 2), of which ischemic stroke accounts for the majority (3). Ischemic stroke is a multifactorial, complex disease that is a combination of genetic and environmental factors (4–6). Even after good control of stroke risk factors (environmental factors) such as diabetes, hypertension, hyperlipidemia and smoking, stroke still occurs in some people (7), it suggests that genetic factors are crucial in the onset of stroke. In rare cases, stroke can be directly caused by monogenic disease, where a rare mutation in a gene is sufficient to cause disease (8). However in most cases, genetic risk factors contribute to the risk for stroke as part of a multifactorial susceptibility (9). Genome-wide association studies identified the risk loci for ischemic stroke and its subtypes related to atrial fibrillation (*PITX 2* and *ZFHX 3*), coronary artery disease (*ABO*, *chr9p21*, *HDAC 9* and *ALDH 2*), blood pressure (*HLDH 2* and *HDAC 9*), pericyte and smooth muscle cell development (*FOXF 2*), coagulation (*HABP2*), carotid plaque formation (*MMP 12*), and neuroinflammation (*TSPAN2*) (10). A more precise strategy is to construct a genetic risk score for ischemic stroke and combining the genetic risk score with risk factor profile and clinical information may eventually lead to better risk prediction (11). Therefore, identifying the etiology of stroke, including genetic etiology, is of great significance for preventing stroke. But, so far, the impact of genes on stroke is not fully understood.

Atherosclerosis is the most prominent cause and risk factor of ischemic stroke. Atherosclerosis is a chronic inflammatory process. Chronic inflammation and vascular endothelial injury play an important role in the initiation and development of atherosclerosis. Chronic inflammation and vascular endothelial function are encoded and regulated by related genes, mutations or polymorphisms of these regulatory genes may impact the occurrence and development of atherosclerosis, and thus affect the susceptibility to stroke. Gardener et al. (12) studied 197 SNPs of 48 inflammatory and endothelial function related genes in 287 patients, and found that 10 genes were closely related to carotid atherosclerosis, revealing the role of inflammatory and endothelial function related genes in the development of atherosclerosis. However, the number of cases in this study was small, and no prospective follow-up was conducted on the cases. The impact of these gene gene interactions and gene environment interactions on atherosclerosis and stroke was not discussed. Therefore, our research group selected these 10 genes, and the 19 variants to further discuss the relationship with atherosclerosis and stroke. Our previous research has confirmed that some inflammation and endothelial genes are related to carotid atherosclerosis (13). However, further study is required to determine whether these genes are associated with ischemic stroke.

Based on the China National Stroke Screening Survey (CNSSS) program, which has been expounded in our previous research (14), we conducted this study: (1) Comparison of general information between ischemic stroke patients at high-risk stroke populations and healthy control group; (2) the relationship between 19 SNPs in genes related to endothelial function and inflammation and stroke, as well as the impact of gene–gene interaction between the 19 SNPs on stroke. In general, these findings are pivotal for identifying the genetic causes of ischemic stroke and contribute to better prevention of cerebrovascular events.

## 2 Materials and methods

### 2.1 Study population

This community-based multicenter cross-sectional survey was part of the CNSSS approved by the Chinese Stroke Screening and Prevention Committee (Grant No. 2011BAI08B01) (15). The research plan was checked and consented by the Ethics Committee of the involved hospitals (the Affiliated Hospital of Southwest Medical University, the People’s Hospital of Deyang City, and Suining Central Hospital). Prior to enrollment, written informed consent forms were acquired from all participants.

The review and organization of this survey can be discovered in past publications by our team (14–16). In short, we randomly selected 8 communities in Sichuan from May 2015 to September 2015 and conducted a structured face-to-face questionnaire survey on residents aged≥40 years who had lived in the communities for over 6 months. The questionnaire includes detailed information about demographic characteristics, history of chronic diseases (such as hypertension, diabetes, dyslipidemia, and atrial fibrillation), behavioral factors, physical examination, and family and personal history of stroke.

### 2.2 Assessment of risk factors and definition of high-risk stroke populations

We evaluated eight common risk factors, including hypertension, diabetes mellitus, coronary artery disease, dyslipidemia, overweight/obesity, smoking, lack of exercise, and stroke family history. We have described the detailed diagnostic criteria in previous studies (16). If these individuals have at least three of the eight conventional risk factors for stroke mentioned above, or have a history of stroke, they are defined as a high-risk stroke population (14–16).

Determine stroke history through self-report and neuroimaging examinations (computed tomography or magnetic resonance imaging) (14). We selected ischemic stroke patients as the research subjects among high-risk populations for stroke, and adopted a 1:1 matching method to select healthy people with a 2-year age difference and the same gender and hypertension as the control group.

### 2.3 Data cleaning procedures

**Figure 1:**
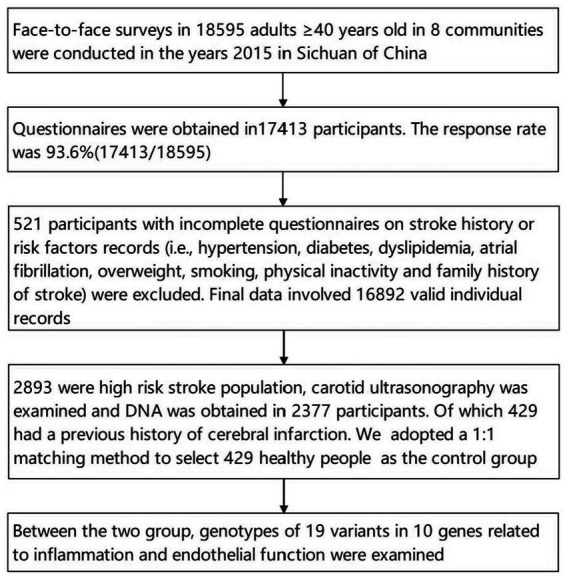
Flow chart in this study.

Out of 16,892 participants, 2,893 individuals were defined as high-risk stroke populations, in high-risk stroke populations, 429 had a previous history of ischemic stroke. The detailed program is shown in Figure 1.

### 2.4 Genotyping

Nineteen SNPs in 10 genes concerned with endothelial function and inflammation were obtained from NCBI database^1^ following the standard: (1) the variants might cause amino acid changes; (2) the variants have been evaluated in the past studies (12, 17); (3) the minor allele frequency > 0.05; (4) nonsynonymous variants.

DNA was extracted from peripheral blood using an modified phenol/chloroform method (17, 18), and genotypes of the 19 SNPs were assessed by using matrix-assisted laser desorption/ionization time of flight mass spectrometry method. In brief, each SNP gene possessed a specific genotype, with two amplification primers and one extension primer. The reaction mix was desalted by adding 6 mg of cation exchange resin (Sequenom Inc., San Diego, CA), mixed, and resuspended in 25 μL of water. Once the primer extension reaction was completed, the samples were spotted onto a 384-well spectroCHIP (Sequenom Inc., San Diego, CA) using a MassARRAY Nanodispenser (Sequenom Inc., San Diego, CA) and genotyped using the MALDI-TOF mass spectrometer. Genotyping was performed in real time with MassARRAY RT software, version 3.0.0.4, and analyzed using the MassARRAY Typer software, version 3.4 (Sequenom Inc., San Diego, CA). The investigators were unaware the clinical data of participants.

### 2.5 Statistical analysis

The data were analyzed using SPSS 22.0 (SPSS Inc. New York, New York, USA). Continuous variables are described as median and interquartile intervals, while categorical variables are described as percentages. Intergroup differences in the baseline characteristics and genotype distributions of the 19 SNPs were evaluated by χ^2^ test or Fisher’s exact test (categorical variables) and Nonparametric tests (continuous variables).

Use the χ^2^ test to evaluate the allele frequency of Hardy–Weinberg equilibrium. Gene–gene interactions between the 19 SNPs were analyzed using generalized multifactor dimensionality reduction (GMDR) method (19), as we previously described (17, 18). We used multivariate logistic regression analysis to assess the stroke risk associated with high-risk interacting genotypes and reported the hazard ratio (HR) with a 95% confidence interval (CI). Input variables with statistical significance when the *p* value < 0.05 in univariate analysis into multivariate logistic regression analysis for adjustment. All tests are 2 sided, *p* value < 0.05 was supposed to statistically significant.

## 3 Results

### 3.1 Baseline characteristics of ischemic stroke patients and healthy control group

Among the 2,893 high-risk stoke populations, 429 had a history of ischemic stroke. Analysis found no statistically significant differences in age, gender, hypertension, BMI, and smoking history between the two groups (Table 1). Compared with the healthy group, ischemic stroke group has a higher proportion of diabetes, heart disease, dyslipidemia, stroke family history, and a higher proportion of lack of exercise (Table 1).

**Table 1:** Baseline characteristics of ischemic stroke patients and healthy control group.

Variables	Stroke patients (*n* = 429)	Healthy group (*n* = 429)	*p*-value
Age (years)	64.0 (58.0–71.0)	64.0 (58.0–69.0)	0.923
Body mass index (kg/m^2^)	26.7 (25.0–27.0)	25.3 (24.1–28.1)	0.155
Male (*n*, %)	158 (36.8%)	158 (36.8%)	1.000
Hypertension (*n*, %)	246 (57.3%)	246 (57.3%)	1.000
Diabetes mellitus (*n*, %)	94 (21.9%)	59 (13.8%)	0.002
Coronary artery disease (*n*, %)	60 (14.0%)	39 (9.1%)	0.025
Smoking history (*n*, %)	104 (24.2%)	128 (29.8%)	0.065
Dyslipidemia (*n*, %)	108 (25.2%)	69 (16.1%)	0.001
Lack of exercise (*n*, %)	251 (58.5%)	185 (43.1%)	0.000
Stroke family history (*n*, %)	55 (12.8%)	23 (5.4%)	0.000

### 3.2 Genotype distributions in objects with and without ischemic stroke

The genotype distributions of the 19 SNPs evaluated in this study were in Hardy–Weinberg equilibrium (*p* value > 0.05). Three genes involved in inflammation and endothelial function were associated with ischemic stroke (*HABP2* rs7923349, *NOS2A* rs8081248, *HABP2* rs932650, Table 2).

**Table 2:** Genotype distributions in objects with and without ischemic stroke.

	Stroke patients (*n* = 429)	Healthy group (*n* = 429)	*p*-value
*TNFSF4* (rs11811788)			0.100
CC	356 (83.00)	362 (84.40)	
CG	68 (15.90)	67 (15.60)	
GG	5 (1.20)	0 (0.00)	
*TNFSF4* (rs1234313)			0.396
AA	188 (43.80)	187 (43.60)	
AG	195 (45.50)	207 (48.30)	
GG	46 (10.70)	35 (8.20)	
*IL6R* (rs1386821)			0.670
GG	1 (0.20)	1 (0.20)	
GT	33 (7.70)	25 (5.80)	
TT	395 (92.10)	403 (93.90)	
*L1A* (rs1609682)			0.458
GG	198 (46.20)	200 (46.60)	
GT	190 (44.30)	198 (46.20)	
TT	41 (9.60)	31 (7.20)	
*IL1A* (rs1800587)			0.055
AA	2 (0.50)	2 (0.50)	
AG	63 (14.70)	41 (9.60)	
GG	364 (84.80)	386 (90.00)	
*TLR4* (rs1927911)			0.953
AA	68 (15.90)	65 (15.20)	
AG	210 (49.00)	210 (49.00)	
GG	151 (35.20)	154 (35.90)	
*ITGA2* (rs1991013)			0.270
AA	196 (45.70)	193 (45.00)	
AG	197 (45.90)	186 (43.40)	
GG	36 (8.40)	50 (11.70)	
*NOS2A* (rs2297518)			0.426
AA	14 (3.30)	12 (2.80)	
AG	115 (26.80)	132 (30.80)	
GG	300 (69.90)	285 (66.40)	
*VCAM1* (rs2392221)			0.248
CC	301 (70.20)	321 (74.80)	
CT	114 (26.60)	99 (23.10)	
TT	14 (3.30)	9 (2.10)	
*TNF* (rs3093662)			0.145
AA	399 (93.00)	409 (95.30)	
AG	30 (7.00)	20 (4.70)	
*VCAM1* (rs3783615)			
AA	429 (100.00)	429 (100.00)	
*PPARA* (rs4253655)			0.624
AG	3 (0.70)	1 (0.20)	
GG	426 (99.30)	428 (99.80)	
*PPARA* (rs4253778)			1.000
CG	1 (0.20)	1 (0.20)	
GG	428 (99.80)	428 (99.80)	
*IL6R* (rs4845625)			0.295
CC	87 (20.30)	106 (24.70)	
CT	216 (50.30)	202 (47.10)	
TT	126 (29.40)	121 (28.20)	
*ITGA2* (rs4865756)			0.454
AA	29 (6.80)	35 (8.20)	
AG	167 (38.90)	151 (35.20)	
GG	233 (54.30)	243 (56.60)	
*TLR4* (rs752998)			0.790
GG	303 (70.60)	294 (68.50)	
GT	116 (27.00)	125 (29.10)	
TT	10 (2.30)	10 (2.30)	
*HABP2* (rs7923349)			0.001
GG	227 (52.90)	198 (46.20)	
GT	164 (38.20)	212 (49.40)	
TT	38 (8.90)	19 (4.40)	
*NOS2A* (rs8081248)			0.000
AA	52 (12.10)	24 (5.60)	
AG	184 (42.90)	168 (39.20)	
GG	193 (45.00)	237 (55.20)	
*HABP2* (rs932650)			0.000
CC	44 (10.30)	2 (0.50)	
CT	183 (42.70)	97 (22.60)	
TT	202 (47.10)	330 (76.90)	

### 3.3 Gene–gene interactions between the 19 variants

We evaluated the relationship between high-order interactions of 19 variants and ischemic stroke using GMDR method. Significant gene–gene interaction was discovered in the 19 variants, and the optimal interaction model for ischemic stroke is the interaction between *HABP2* rs7923349 and *HABP2* rs932650, in which the sign test was 10 and cross-validation consistency was 10/10 (*p* = 0.001; Table 3). The *p*-value of the prediction error based on permutation test is 0.016.

**Table 3:** GMDR analysis of the best models, prediction accuracies, cross-validation consistencies, and *p*-values for ischemic stroke.

Best model*	Training balanced accuracy	Testing balanced accuracy	Cross-validation consistency	Sign test (*p*-value)
1	0.6492	0.6492	10/10	10 (0.0010)
1, 2	0.6678	0.6677	10/10	10 (0.0010)
1, 2, 3	0.6854	0.6725	9/10	10 (0.0010)
1, 2, 3, 4	0.7034	0.6415	5/10	10 (0.0010)
1, 2, 3, 5, 6	0.7370	0.6078	4/10	10 (0.0010)
1, 3, 5, 6, 7, 8	0.7868	0.5741	5/10	9 (0.0107)
1, 3, 4, 5, 6, 7, 8	0.8491	0.5820	9/10	10 (0.0010)
1, 2, 3, 4, 5, 6, 7, 8	0.9021	0.5400	5/10	9 (0.0107)
1, 2, 3, 4, 5, 6, 7, 8, 9	0.9434	0.5615	8/10	6 (0.3770)
1, 2, 3, 4, 5, 6, 7, 8, 9, 10	0.9671	0.5400	6/10	7 (0.1719)
1, 2, 3, 4, 5, 6, 7, 8, 9, 10, 11	0.9821	0.6216	10/10	8 (0.0547)
1, 2, 3, 4, 5, 6, 7, 8, 9, 10, 11, 12	0.9877	0.6150	5/10	8 (0.0547)
1, 2, 3, 4, 5, 6, 7, 8, 9, 10, 11, 12, 13	0.9917	0.6681	5/10	9 (0.0107)
1, 2, 3, 4, 5, 6, 7, 8, 9, 10, 11, 12, 13, 14	0.9938	0.7636	10/10	9 (0.0107)
1, 2, 3, 4, 5, 6, 7, 8, 9, 10, 11, 12, 13, 14, 15	0.9938	0.7356	10/10	9 (0.0107)
1, 2, 3, 4, 5, 6, 7, 8, 9, 10, 11, 12, 13, 14, 15, 16	0.9938	0.6689	10/10	9 (0.0107)
1, 2, 3, 4, 5, 6, 7, 8, 9, 10, 11, 12, 13, 14, 15, 16, 17	0.9938	0.6689	10/10	9 (0.0107)
1, 2, 3, 4, 5, 6, 7, 8, 9, 10, 11, 12, 13, 14, 15, 16, 17, 18	0.9938	0.6667	10/10	9 (0.0107)
1, 2, 3, 4, 5, 6, 7, 8, 9, 10, 11, 12, 13, 14, 15, 16, 17, 18, 19	0.9938	0.6667	10/10	9 (0.0107)

GMDR, generalized multifactor dimensionality reduction. *Numbers 1–19 represent rs932650, rs7923349, rs8081248, rs1609682, rs1234313, rs4845625, rs1927911, rs4865756, rs1991013, rs2297518, rs2392221, rs752998, rs1386821, rs11811788, rs1800587, rs3093662, rs3783615, rs4253655, and rs4253778, respectively.

### 3.4 Different genotype combinations and the risk of ischemic stroke

Subsequently, the correlation between different genotype combinations and stroke risk in two interacting variants were evaluated. It was found that compared with the individuals with wild-type genotype of the two variants (rs7923349 GG, rs932650 TT), five genotype combinations associated with a higher risk of ischemic stroke, including rs7923349 TT, rs932650 TT (OR = 3.53, 95% CI: 1.78–6.99, *p* = 0.000); rs7923349 GT, rs932650 CC (OR = 21.28, 95% CI: 2.76–166.67, *p* = 0.000); rs7923349 GT, rs932650 CT (OR = 2.41, 95% CI: 1.55–3.75, *p* = 0.000); rs7923349 GG, rs932650 CC (OR = 50.00, 95% CI: 6.85–333.33, *p* = 0.000); rs7923349 GG, rs932650 CT (OR = 4.00, 95% CI: 2.58–6.21, *p* = 0.000) (Table 4). We defined the above five genotype combinations as high-risk interaction genotypes, while other genotype combinations that did not reach statistical significance (*p* > 0.05) are considered low-risk interaction genotypes.

**Table 4:** Different genotype combinations and the risk of ischemic stroke.

rs7923349	GG	TT	GT	GT	GG	GG	TT	GT
rs932650	TT	TT	CC	CT	CC	CT	CT	TT
OR	1*	3.53	21.28	2.41	50.00	4.00	2.64	0.80
95%CI	-	1.78–6.99	2.76–166.67	1.55–3.75	6.85–333.33	2.58–6.21	0.84–8.33	0.55–1.16
*P*-value	-	0.000	0.000	0.000	0.000	0.000	0.086	0.237

*Using wild-type genotype as a reference OR. OR, odds ratio; CI, confidence interval.

### 3.5 Correlation between high-risk interactive genotypes and ischemic stroke

There were 249 carriers of the high-risk interactive genotypes among 429 ischemic stroke patients, 180 carriers in healthy control group. The proportion of high-risk genes carried by ischemic stroke patients is higher than that of healthy individuals (58.0% [249/429] vs. 25.2% [108/429], χ^2^ = 95.372, *p* < 0.001).

In addition, we used multivariate logistic regression to evaluate the risk of ischemic stroke associated with high-risk interaction genotypes between *HABP2* rs7923349 and *HABP2* rs932650. The high-risk interactive genotypes were assigned as one, while the low-risk interactive genotypes were assigned as zero. Other variables that showed a significant correlation (*p* < 0.05) with ischemic stroke in univariate analysis were adjusted by inputting them into the multivariate logistic regression model. The results showed that after adjusting for covariates, high-risk interaction genotypes in *HABP2* rs7923349 and *HABP2* rs932650 were independently associated with higher stroke risk (OR, 3.578, 95% CI: 2.618–4.890, *p* < 0.001,Table 5). Furthermore, we used the H-L test to judge the goodness of fit of the multivariate logistic regression model, and the results showed that the model had a good fit (χ2value = 13.100, *p* = 0.108).

**Table 5:** Association between high-risk interactive genotypes and ischemic stroke.

Risk factor	OR	95% CI	*p*-value
Stroke family history	2.712	1.563–4.705	0.000
Diabetes mellitus (*n*, %)	1.577	1.059–2.350	0.025
Coronary artery disease (*n*, %)	1.588	0.988–2.555	0.056
Smoking history (*n*, %)	0.817	0.585–1.143	0.238
Dyslipidemia (*n*, %)	1.401	0.959–2.048	0.081
Lack of exercise (*n*, %)	1.808	1.339–2.442	0.000
*HABP2* rs7923349 GG	1.307	0.968–1.764	0.081
*NOS2A* rs8081248 AA	2.575	1.488–4.457	0.001
*HABP2* rs932650 CC	11.110	2.605–47.379	0.001
High-risk interactive genotype	3.578	2.618–4.890	0.000

## 4 Discussion

In this study, we selected 429 patients with a history of ischemic stroke from the high-risk stroke populations as the experimental group, and matched 429 healthy controls with similar age, gender, and hypertension history. Compared with the control group, the experimental group has a higher proportion of diabetes, heart disease, dyslipidemia, stroke family history, and a higher proportion of lack of exercise. In single SNP analysis, we found that two endothelial function related gene variants (*HABP2* rs7923349, *HABP2* rs932650) and one inflammation related gene variant (*NOS2A* rs8081248) were significantly associated with ischemic stroke. Moreover, GMDR analysis showed significant gene–gene interactions between *HABP2* rs7923349, *HABP2* rs932650, and high-risk interaction genotypes between these two variants were independently associated with higher stroke risk.

Genetics has been discovered to play an important role in the initiation of stroke. In large-scale genomic research, multiple genetic associations have been found between various risk factors and ischemic stroke itself (20, 21). Nevertheless, the exact mechanism and etiology of stroke development are very complex and have not been fully described yet (22). Our team’s previous research has shown that endothelial and inflammatory genes are relate to carotid atherosclerosis (13), this study aims to investigate whether endothelial and inflammatory genes are associated with ischemic stroke. As far as we know, our study is the first to investigate the possible association between genetic variations related to inflammation and endothelial function and ischemic stroke in the Chinese population.

Inflammation plays a vital role in the increase of inflammatory cell migration and the initiation of atherosclerosis (23, 24). Inflammatory gene polymorphism may affect the progression and development of atherosclerosis through direct or indirect interaction with vascular risk factors (25). The inducible nitric oxide synthase (iNOS) encrypted by the *NOS2A* gene is one of the important inflammatory mediators released by macrophages (26), the NO catalyzed by it can react with peroxy anion to form peroxynitrite, which can cause endothelial damage, promote the inflammatory reaction of vascular wall, and promote the progress and development of atherosclerosis. In addition, the expression of iNOS can induce the expression of matrix metalloproteinase 9 gene (matrix metalloproteinase, MMP-9) (27), MMP-9 can decompose collagen components in atherosclerotic plaque, leading to instability of atherosclerotic plaque and eventually leading to ischemic stroke. Our previous research has also shown that *NOS2A* is associated with unstable plaques (17). In this study, The *NOS2A* gene is associated with stroke, implying its essential role in different stages of stroke.

Endothelial function control platelet adhesion and aggregation, interactions between platelets and immune cells, capillary tension, and adhesion between endothelial cells to maintains the vascular barrier. Endothelial dysfunction may damage the integrity of blood vessels and is related to diverse human diseases, for instance coronary artery disease, atherosclerosis and stroke (28). The hyaluronic acid binding protein 2 (*HABP2*) gene encodes a cell adhesion protein (hyaluronic acid binding protein 2) that regulates vascular integrity. This gene may be a genetic susceptibility locus for stroke (29), which is concordance with this study.

Stroke is a sophisticated disease because it does not follow Mendelian inheritance patterns, which may be the outcome of gene–gene interactions (17, 18). Single gene methods may not be effective in identifying the genetic causes of complex diseases, therefore evaluating gene gene interactions is necessary for studying the genetic mechanisms of stroke. In this study, we used GMDR analysis to assess the gene–gene interactions between 19 variants and ischemic stroke. The most remarkable finding in our study was the significant gene–gene interaction between *HABP2* rs7923349 and *HABP2* rs932650. High-risk interaction genotypes between these two variants were independently related to higher stroke risk, indicating that the synergistic interaction between these two variants leads to ischemic stroke. GMDR analysis emphasizes the complexity of genetic effects and the potential synergistic effects of variants in increasing stroke risk. In addition, past studies have also explored the roles of different genes in stroke (18, 30). Nevertheless, the molecular mechanisms underlying the interaction between these two variants are still unclear. One possible illustration is that these two variants can encode and regulate endothelial function related enzymes, which participate in the important pathogenic mechanisms of ischemic stroke. Therefore, further research is needed to explore the molecular mechanisms underlying the interactions between these two variants.

Although our findings are encouraging, but there has several limitations. First, our study was a case–control study, we choose patients with ischemic stroke in the high-risk populations and a healthy control group. Therefore, there may be selection bias, and because it is a retrospective study, due to self-reported questionnaires, there may be recall bias. The small sample size and the selection of high-risk stroke individuals based on predefined criteria may exclude participants with atypical presentations or less common risk factors, potentially leading to skewed results. Our research results can only suggest a possible association between genes and stroke. Because this study is a retrospective study, it is difficult to obtain all the examination results of stroke patients, so it is difficult to distinguish various stroke types, therefore the etiology of our patients included atherosclerosis, arteriolar occlusion, and perhaps a small number of unknown causes. Prospective research is needed in the future to explore the genetic risk factors in different etiologies of stroke. Second, this study only conducted a sampling survey on residents aged ≥40 years; as a result, our conclusion cannot be extended to all populations in southwestern China. Third, although we have examined the role of several important genes related to endothelial function and inflammation, there are still some known and unknown genes that have not been studied. Moreover, the molecular mechanism of gene interaction was not further explored in this study. In the future, more research involving genetic variations should be conducted to further elucidate the impact of gene–gene interactions on ischemic stroke.

## 5 Conclusion

In this study, we identified the associations of variants in *HABP2* rs7923349, *HABP2* rs932650, *NOS2A* rs8081248 with stroke. There was an obvious gene–gene interaction observed in *HABP2* rs7923349, *HABP2* rs932650, the high-risk interaction genotype between the two variants is an independent risk factor for ischemic stroke. According to our research results, active intervention in traditional risk factors, such as dyslipidemia and diabetes mellitus, may be very important to reduce the risk of stroke in high-risk stroke population with high-risk interactive genotypes. In addition the gene–gene interactive analysis used in our study may be very beneficial in elucidating the sophisticated genetic risk factors for ischemic stroke. Further research exploring the molecular mechanisms underlying interactions between genetic variants is essential to deepen our understanding of the genetic mechanisms underlying stroke.

## Data Availability

The datasets presented in this study can be found in online repositories. The names of the repository/repositories and accession number(s) can be found in the article/Supplementary material.

## Footnotes

^1^ https://www.ncbi.nlm.nih.gov/snp
